# Annals of General Psychiatry reviewer acknowledgement 2012

**DOI:** 10.1186/1744-859X-12-5

**Published:** 2013-04-05

**Authors:** Konstantinos N Fountoulakis

**Affiliations:** 1 Department of Psychiatry, School of Medicine, Aristotle University of Thessaloniki, Thessaloniki, Pylaia 55535, Greece

## Abstract

**Contributing reviewers:**

The editors of *Annals of General Psychiatry* would like to thank all of our reviewers who have contributed to the journal in volume 11 (2012).

## 

Nobuhito Abe

Japan

Tasnime Akbaraly

France

Ramona Alaggia

Canada

Umberto Albert

Italy

William Apfeldorf

United States of America

Jean-Michel Azorin

France

Daryl Bainbridge

Canada

Thomas Barnes

United Kingdom

Robert Berman

United States of America

Carlo Blanco

United States of America

Julia Boyle

United Kingdom

David Brent

United States of America

Edson Sherwood Brown

United States of America

Lisa Buchy

Canada

Oscar Bukstein

United States of America

Monika Bullinger

Germany

Rosetta Cardone

Italy

Franco Cavallo

Italy

Ka Fai Chung

Hong Kong

Young-Chul Chung

South Korea

Lars Clemmensen

Denmark

Carla Crespo

Portugal

Rudi Crncec

Australia

Serdar Dursun

Canada

Chad Ebesutani

United States of America

Marc-Andreas Edel

Germany

Dean Elbe

Canada

Betina Elfving

Denmark

Peter Enticott

Australia

Carlo Faravelli

Italy

Giovanni Fava

Italy

Maria Luisa Figueira

Portugal

Kishore Gadde

United States of America

Beata Galińska-Skok

Poland

Marta Ghisi

Italy

George Giannakopoulos

Greece

Michael Gitlin

United States of America

Renee Goodwin

United States of America

Daniela Goursand

Brazil

Philip Harvey

United States of America

Kotaro Hatta

Japan

Negoro Hideki

Japan

Qin Jiang

United States of America

Hironobu Katsuyama

Japan

Ikuko Kishida

Japan

Michael Koelch

Germany

Leena Koivusilta

Finland

David Levitt

United States of America

Matthias Liechti

Switzerland

Kamala London

United States of America

Antonio Madueno

Spain

Arja Mainio

Finland

Mario Maj

Italy

Ferenc Martenyi

Austria

Almudena Martorell

Spain

Valentin Mbekou

Canada

Christina McCrae

United States of America

Mario Mendez

United States of America

Scott Moeller

United States of America

Somaia Mohamed

United States of America

Brent Moore

United States of America

Gunnar Morken

Norway

Martin Mumenthaler

United States of America

Jackob Najman

Australia

Hiroyuki Nakamura

Japan

Tomohiro Nakao

Japan

Urs Nater

Germany

David Neubauer

United States of America

Takashi Okada

Japan

Andreas Pavalkis

Cyprus

Vassilis Pavlopoulos

Greece

Juan D Pedrera-Zamorano

Spain

Leah Phillips

Canada

Maurizio Pompili

Italy

Dina Popovic

Spain

Rajeev Ramchand

United States of America

Sathyanarayana T S Rao

India

Riju Ray

Belgium

Zoltan Rihmer

Hungary

Nicolas Rohleder

United States of America

Soledad Romero

Spain

Janusz Rybakowski

Poland

Seija Sandberg

United Kingdom

Junji Saruwatari

Japan

Satoshi Sasaki

Japan

Philip Seeman

Canada

Kang Sim

Singapore

Rhonda Small

Australia

Cheryl So

Hong Kong

Erkki Soini

Finland

Jan Sorensen

Denmark

Justin Springer

United States of America

Mary Stewart

United Kingdom

Yoshihiro Tadori

Japan

Hidehiko Takahashi

Japan

Michihiro Takahashi

Japan

Takeshi Terao

Japan

Anne Thorup

Denmark

Albina Torres

Brazil

Chi-Chuan Wang

Taiwan

Janet Williams

United States of America

Lutz Wittmann

Switzerland

Klaus Wölfling

Germany

Guohua Xia

United States of America

Hakuei Yamashita

Japan

Toshiyuki Yasui

Japan

Dimitrios Zavras

Greece

